# Lupine seed allergy in a patient after liver transplantation diagnosed by component resolved diagnosis

**DOI:** 10.1186/2045-7022-3-S3-P23

**Published:** 2013-07-25

**Authors:** S Belohlavkova, M Fuchs

**Affiliations:** 1 Immuno-flow, Prague, Czech Republic

## Background

Component resolved diagnosis (CRD) is becoming a powerful tool in food allergy diagnostics. It helps to distinguish, whether positivity of specific IgE relates to clinically relevant allergy, or only to sensitisation because of cross-reactivity.

## Methods

We present a case history of 4,5 years old girl investigated because of severe diarrhoea and malnutrition. The child has been diagnosed with biliary duct atresia with porto-entero anastomosis being created in her age of 7 days. At the age of 4 years, she has been examined elsewhere for seasonal complaints and she was diagnosed with grass allergy. She has had detected positivity of specific IgE against milk, egg and wheat flour and based on these findigs she was recommended a gluten free diet.

## Results

However, during following months, she was suffering of severe diarrhoea, liver failure progressed and she had to undergo liver transplantation. Diarrhoea and malnutrition continued despite strictly followed gluten free diet. She had undergone another allergology testing using CRD and suprisingly, sensitisation to vicilin family (Ara h1) and conglutins (Ara h 2) has been detected. She was eating mainly gluten free flour with high content of lupine at that time. Assuming high probability of lupine allergen cross-reactivity with allergens of seed storage protein families (including vicilins and conglutins), we added skin prick tests with lupine seed with positive result. Thereafter, the diet was reevaluated. After a strict prohibition of Ara h1 and Ara h 2 homologous proteins (mainly lupine) her condition markedly improved. Gluten flours were included in her diet without any complaints. Currently, the girl is doing well.

## Conclusion

To recommend gluten free diet only based on positivity of specific IgE antibodies can be occasionally not only useless, but also harmful. A significant proportion of gluten free diet consists of flours with legumes, mainly the lupine. In cases of sensitisation to seed storage proteins may clinically significant crossr-reactivity with lupine worsen the situation. Current case demonstrates, how CRD diagnostics helped to detect the main trigger point of severe gastrointestinal food allergy.

## Disclosure of interest

None declared.

